# Ergonomics in the operating room

**DOI:** 10.1007/s00464-016-5247-5

**Published:** 2016-10-17

**Authors:** Shiromani Janki, Evalyn E. A. P. Mulder, Jan N. M. IJzermans, T. C. Khe Tran

**Affiliations:** Division of HPB and Transplant Surgery, Erasmus MC, Department of Surgery, University Medical Center, Room no. H-822k, ’s-Gravendijkwal 230, 3015 CE Rotterdam, The Netherlands

**Keywords:** Ergonomics, Musculoskeletal complaints, Surgeons, Therapy, Work absence

## Abstract

**Background:**

Since the introduction of minimally invasive surgery, surgeons appear to be experiencing more occupational musculoskeletal injuries. The aim of this study is to investigate the current frequency and effects of occupational musculoskeletal injuries on work absence.

**Methods:**

An online questionnaire was conducted among all surgeons affiliated to the Dutch Society for Endoscopic Surgery, Gastrointestinal Surgery, and Surgical Oncology. In addition, this survey was conducted among surgeons, gynaecologists, and urologists of one cluster of training hospitals in the Netherlands.

**Results:**

There were 127 respondents. Fifty-six surgeons currently suffer from musculoskeletal complaints, and 30 have previously suffered from musculoskeletal complaints with no current complaints. Frequently reported localizations were the neck (39.5 %), the erector spinae muscle (34.9 %), and the right deltoid muscle (18.6 %). Most of the musculoskeletal complaints were present while operating (41.8 %). Currently, 37.5 % uses medication and/or therapy to reduce complaints. Of surgeons with past complaints, 26.7 % required work leave and 40.0 % made intraoperative adjustments. More surgeons with a medical history of musculoskeletal complaints have current complaints (OR 6.1, 95 % CI 1.9–19.6). There were no significant differences between surgeons of different operating techniques in localizations and frequency of complaints, or work leave.

**Conclusions:**

Despite previous various ergonomic recommendations in the operating room, the current study demonstrated that musculoskeletal complaints and subsequent work absence are still present among surgeons, especially among surgeons with a positive medical history for musculoskeletal complaints. Even sick leave was necessary to fully recover. There were no significant differences in reported complaints between surgeons of different operating techniques. Almost half of the respondents with complaints made intraoperative ergonomic adjustments to prevent future complaints. The latter would be interesting for future research.

Minimally invasive surgery acclaims several advantages for the patient, such as less postoperative pain, shorter recovery time, and better cosmetic results [1]. After the first laparoscopic cholecystectomy in 1987, minimally invasive surgery has been widely accepted and often the first choice for many surgical procedures [2–4]. Although there is evident benefit for patients by this operating technique [1], surgeons appear to be experiencing more occupational musculoskeletal injuries [5–8]. An occupational injury is any injury to the body incurred in the work environment [9].

Freedom of movement during minimally invasive surgery is more restricted compared to open surgery [10, 11]. The disadvantages of minimally invasive surgery are a static pose of neck and back for a long period to stay in place and look at the monitor, and frequently uncomfortable positioning of arms and shoulders to optimize camera position and minimize movements within the operation field [7, 12]. Maintenance of the static posture for prolonged periods of time is the source of increased muscle fatigue [11, 13]. However, both open and endoscopic operating techniques require a non-ergonomic posture. Open surgical procedures are characterized by a head-bent and back-bent posture. In order to balance the upper body, increased muscle activity is required [14]. Surgeons suffering from musculoskeletal injuries seem to require more analgesics, including non-steroid anti-inflammatory drugs and muscle relaxants, and/or therapy, such as massage therapy or physical therapy [15–17]. The complaints can sometimes lead to sick leave or even temporary resign of surgical tasks [17, 18].

Surgeons’ non-ergonomic posture during minimally invasive operations is becoming increasingly known. Over the years, these insights have led to ergonomic guidelines. These guidelines have led to ergonomic proposals in the operating theatre during minimally invasive surgery. Firstly, two or more monitors should be present, which are adjustable in height to prevent discomfort in the neck [19, 20]. Secondly, special features of endoscopic instruments led to non-ergonomic position of the wrist and fingers and adjusted redesigned instruments should be the preferred choice [18]. Thirdly, a mismatch between table height and the surgeon’s height increases muscular strain; thus, an optimal operation table height should be pursued [21, 22]. Lastly, a lack of balance can occur when using foot pedals by movement of bodyweight to the other foot, resulting in more muscle fatigue. Operating often requires standing, awkward body positions, so a more neutral position should be considered [6, 23]. All these adjustments have resulted in less occupational complaints and reduction in sick leave [24].

Studies have been conducted to evaluate musculoskeletal complaints experienced by surgeons during minimally invasive surgery [7, 25–27]. Unfortunately, these studies contained several limitations regarding lack of demographic information [12, 25], lack of comparative operation techniques and small sample sizes [16, 28–31], and lack of sick leave rate among surgeons. Both lack of awareness and implementation of ergonomic guidelines among surgeons seemed to be the major problem in the occurrence of musculoskeletal complaints [2, 32, 33]. The aim of this study was to gain insight into the current state of musculoskeletal injuries and subsequent frequency and duration of sick leave among surgeons, and whether the ergonomic adjustments have reduced the incidence of these occupational complaints over the years.

## Materials and methods

### Study population

The Dutch Societies for Endoscopic Surgery (NVEC) [34], Gastrointestinal Surgery (NVGIC) [35], and Surgical Oncology (NVCO) [36] are associations consisting of members from different surgical disciplines. Varying from gastrointestinal and oncologic surgeons, to gynaecologists and urologists, both specialized in laparoscopic surgery or other operating techniques.

In the Netherlands, surgical residents have to complete a training programme that lasts 6 years in order to become a certified surgeon, of which 2 years are spent in a university hospital and four in a district hospital. The first four years, training in general surgery is offered. The remaining 2 years consist of specialty training in one of the following subspecialties: gastrointestinal surgery, surgical oncology, trauma surgery, or vascular surgery. This postgraduate training in general surgery is organized in eight training regions, each consisting of one university hospital and several affiliated district general training hospitals [37–39].

### Study design

This survey is a cross-sectional study and was conducted online using Qualtrics (San Francisco, CA, USA). The study population consisted of all members of the NVEC, the NVGIC, and the NVCO. Furthermore, the survey was conducted among surgeons specialized in gastrointestinal surgery or surgical oncology, gynaecologists, and urologists, in one cluster of eight training hospitals. An invitation to fill out the survey was sent on 1st July, 2015. Three weeks later, a reminder was sent to non-responders. The survey was entirely anonymous.

### Online survey

The survey composed of questions based on a thorough review of the literature and was based on three categories: (A) demographics, (B) operating characteristics, and (C) musculoskeletal disorders. A summarized version of the questionnaire is provided in Table 1. The first section contained questions on characteristics of the respondent (age, gender, height and weight, dominant hand, and glove size), years of experience, and a positive or negative medical history for musculoskeletal injuries. The second section comprised questions on surgeons’ operating profile (how many operations/hours per week, operating techniques). In addition, questions on surgeons’ hours of sleep per night, ergonomically work environment outside the operation room (OR), and hours of exercise per week were asked. Part three surveyed details about musculoskeletal disorders with additional questions on timeline, localization and corresponding pain score, use of analgesics and/or therapies, and whether the surgeon was diagnosed with a musculoskeletal disorder, e.g. hernia. Furthermore, questions were asked whether any (ergonomic) adjustments were taken to reduce or prevent complaint(s) and whether surgeons temporarily stopped operating or required sick leave due to their musculoskeletal complaints.

**Table 1:** Summarized version of the questionnaire

A. Demographic and background data
Since when were you certified as a surgeon?
What is your area of specialization?
Where do you work at the moment?
How old are you?
What is your gender?
How tall are you? (cm)
How much do you weigh? (kg)
What is your dominant hand?
What is your glove size?
What is your medical history concerning the musculoskeletal system

B. Operating characteristics
How many operations do you perform weekly?
How many of these operations take longer than 3 h?
On average, how many hours do you operate a week?
Which operating technique do you use (in terms of percentage)?
How many hours do you sleep a night?
Is your working-/consulting room [not the OR] furnished ergonomically?
How many hours do you exercise a week?

C. Musculoskeletal disorders
Are you suffering from musculoskeletal disorders?
*No, never* *→* *End of survey*
*No, but I did in the past/Yes*
When did these complaints start?
Please click on the concerning body parts
From which part do/did you suffer the most?
How do you/would you’ve administered the pain score on this disorder, using the VAS score?
Do/Did you take analgesics against the complaint(s)?
Do/Did you use any additional treatments (such as physiotherapy, massage) in order to reduce the disorder(s)?
*Please specify*
When do/did the complaint(s) appear?
Did a medical specialist diagnose you based on this/these complaint(s)?
*Please specify*
Does your employee know about your musculoskeletal complaint(s)?
Have you ever taken measures or adjustments in order to reduce this/these complaint(s)?
*Please specify*
Have you ever stopped operating/required sick leave (temporarily) due to these musculoskeletal complaints?
*Please specify*

### Study outcome parameters

The main study parameter was frequency and duration of work absence due to occupational musculoskeletal complaints. The secondary study parameters were localization and frequency of musculoskeletal complaints and the use of medication and/or therapy.

### Statistical analysis

Descriptive analyses were used to define respondent characteristics. Musculoskeletal complaints were assessed for the years of experience, operating techniques, number of operating hours, number of operations, hours of sleep, and hours of physical activity. Continuous variables were compared with the Mann–Whitney *U* test and categorical variables with the Pearson Chi-square test. Statistical analyses were performed using SPSS 23.0 statistical package (IBM SPSS Statistics, Version 23.0. Armonk, NY: IBM Corp.). A *p* value <0.05 (two-sided) was considered statistically significant.

## Results

### Respondent characteristics

The survey was sent to 349 surgeons and was completed online by 127 (36.4 %), including 118 (33.8 %) certified specialists being general surgeons (76.3 %), gynaecologists (12.7 %), urologists (7.6 %), and paediatric surgeons (3.4 %). Most respondents were male (66.9 %). Median age was 47 years (33.0–63.0). The height and weight were a median of 180.0 cm (156.0–204.0) and a median of 79.0 kg (52.0–118.0), respectively. Forty-two respondents worked in an academic hospital (35.6 %) and the others in a district hospital (64.4 %). Overall, respondents had a median of 14.0 years (0.0–33.0) working experience, 102 were right-handed (86.4 %), 6 were left-handed (5.1 %), and 10 were ambidextrous (8.5 %). Glove size was a median of 7.5 (6.0–9.0).

### Musculoskeletal complaints: characteristics

A total of 56 surgeons (47.5 %) currently suffer from musculoskeletal complaints, and 30 surgeons (25.4 %) reported that they have previously suffered from musculoskeletal complaints, but do not have complaints anymore. Table 2 gives an overview of the current demographics of the respondents, categorized by musculoskeletal complaints: never, previous, and current. There were no significant differences in respondent characteristics between respondents with current musculoskeletal complaints compared with respondents who never had complaints for age (*p* = 0.086), gender (*p* = 0.899), years of certification (0.114), height (*p* = 0.829), weight (*p* = 0.522), glove size (*p* = 0.487), or operating hours per week (*p* = 0.263). However, there was a significant difference in a positive medical history for musculoskeletal complaints between these groups, 46.4 % versus 12.5 % (*p* < 0.001), respectively. Respondents with a positive medical history for musculoskeletal complaints were more likely to have current complaints (OR 6.1, 95 % CI 1.9–19.6; *p* = 0.003) or had previous complaints (OR 4.1, 95 % CI 1.1–14.6; *p* = 0.033). Of all respondents with current or previous complaints and a positive medical history, most of their medical history comprised of cervical and/or lumbar hernia (*n* = 17). Other reported medical conditions were shoulder problems, e.g. frozen shoulder, luxation, impingement or a fractured olecranon (*n* = 4), anterior cruciate ligament and/or meniscus injury (*n* = 6), chronic back pain (*n* = 4), facet joint fixation (*n* = 2), Achilles tendon injury (*n* = 1), pelvic pain (*n* = 1), and spinal fusion after spondylodiscitis (*n* = 1). Most of them (73.0 %) reported the same complaints, sometimes accompanied with other pain localizations. In addition, a positive medical history for complaints of the back, knee, and shoulder was more frequently reported by surgeons with current complaints concerning the back (*p* = 0.018), knee (*p* = 0.001), and shoulder (*p* = 0.020), respectively.

**Table 2:** Respondent characteristics—musculoskeletal complaints (never/previous/current)

Characteristics	Never (*n* = 32)	Previous (*n* = 30)	Current (*n* = 56)
Gender (male)	21 (65.5 %)	22 (73.3 %)	36 (64.3 %)
Age (years)	47.0 (33.0–60.0)	49.0 (35.0–63.0)	47.0 (35.0–63.0)
Height (cm)	178.0 (164.0–193.0)	185.5 (167.0–202.0)	180.5 (156.0–204.0)
Weight (kg)	76.5 (62.0–105.0)	79.5 (55.0–110.0)	80.0 (52.0–118.0)
Specialism			
General surgeon	22 (68.8 %)	24 (80.0 %)	44 (78.6 %)
Urologist	4 (12.5 %)	3 (10.0 %)	2 (3.6 %)
Gynaecologist	6 (18.8 %)	2 (6.7 %)	7 (12.5 %)
Paediatric surgeon	0	1 (3.3 %)	3 (5.4 %)
Hospital (academic/district)	10/22 (31.3 %/68.8 %)	8/22 (26.7 %/73.3 %)	24/32 (42.9 %/57.1 %)
Dominant hand			
Right	29 (90.6 %)	24 (80.0 %)	49 (87.5 %)
Left	1 (3.1 %)	3 (10.0 %)	2 (3.6 %)
Ambidextrous	2 (6.3 %)	3 (10.0 %)	5 (8.9 %)
Glove size	7.5 (6.5–8.5)	7.5 (6.5–8.5)	7.5 (6.0–9.0)
Years of practice	13.0 (2.0–25.0)	15.5 (1.0–29.0)	13.0 (0.0–33.0)
Medical history			
Blanco	28 (87.5 %)	19 (63.3 %)	30 (53.6 %)
Operation technique^a^			
Endoscopic	20 (62.5 %)	20 (66.7 %)	34 (60.7 %)
Open	11 (34.4 %)	5 (16.7 %)	17 (30.4 %)
Open = endoscopic	0	5 (16.7 %)	4 (7.1 %)
Robotic	1 (3.1 %)	0	1 (1.8 %)
Hours of sleep (per night)	6.5 (5.0–8.0)	7.0 (6.0–7.5)	6.75 (5.0–8.0)
Hours of exercise (per week)	2.0 (0.0–12.0)	2.0 (0.0–12.0)	2.0 (0.0–12.0

Continuous variables are depicted as median (range). Categorical variables are depicted as absolute values (%)

^a^Most frequently used operating technique

### Musculoskeletal complaints: localization, pain score, and consequences

Table 3 gives an overview of all reported localizations of previous and past complaints by gender, right/left-handed, visual analogue scale (VAS), and treatment. The most frequently reported localization of complaints was the neck (39.5 %), followed by the erector spinae muscle (34.9 %), the right deltoid muscle (18.6 %), and the right latissimus dorsi muscle (17.4 %). There was no difference in most frequently reported side of complaints in the upper extremity between right- and left-handed surgeons (*p* = 0.823). Most of the musculoskeletal complaints were only present while operating (41.8 %), followed by postoperative pain (40.6 %), and 15 surgeons (17.4 %) suffered from continuous pain/discomfort. In total, 18 respondents (20.9 %) reported that their complaints started before becoming a certified surgeon, of which four (22.2 %) reported that the complaints started more than ten years before that. Eight respondents (44.4 %) noted to suffer from these musculoskeletal complaints ever since. One of the respondents explicitly noted that the complaints both started and ended before the start of surgical residency.

**Table 3:** Previous and current complaints

Localization	%	Male/female	Right/left handed	VAS ≤ 5/> 5	Analgesics and/or therapy (yes)^b^
Neck	39.5	18/16	25/4	25/9	14
Erector spinae M.	34.9	21/9	24/3	22/8	15
Lat. dorsi muscle R^a^	17.4	7/8	11/3	13/2	5
Lat. dorsi muscle L^a^	4.7	1/3	2/1	3/1	2
Hips	4.7	1/3	3/0	2/2	2
*Upper extremity R*					
Deltoid muscle	18.6	9/7	12/2	14/2	9
Elbow	3.5	3/0	3/0	2/1	1
Forearm	3.5	1/2	3/0	3/0	1
Wrist	8.1	5/2	5/0	4/3	2
Fingers	5.8	3/2	4/0	4/0	1
Thumb	4.7	3/1	4/0	5/0	3
*Upper extremity L*					
Deltoid muscle	12.8	3/8	9/1	7/4	5
Elbow	5.8	2/3	5/0	3/2	1
Forearm	1.2	0/1	1/0	1/0	1
Wrist	4.7	2/2	2/0	3/1	1
Fingers	8.1	3/4	2/0	2/0	0
Thumb	2.3	2/0	6/0	6/1	2
*Lower extremity R*					
Knee	7.0	5/1	4/0	4/2	1
Ankle/foot	7.0	5/1	5/0	4/2	3
*Lower extremity L*					
Knee	4.7	3/1	3/0	4/0	2
Ankle/foot	3.5	2/1	3/0	2/1	2

^a^ *R* right sided, *L* left sided

^b^Therapy: e.g. massage therapy or physical therapy

In approximately 75 % of the complaints, a VAS of 5 or lower was scored. More than half of the respondents with complaints (57.9 %) reported to use medication and/or therapy to reduce complaints. Currently, 37.5 % uses medication and/or therapy to reduce complaints. More than two different analgesics were taken if a VAS of eight or higher was experienced. The intake of opioids was reported among three surgeons who reported a VAS of nine. A VAS of ten was reported once due to a cervical hernia (C6–C7), for which a cervical plexus block was provided.

In 18.6 % of the musculoskeletal complaints, the employer had been informed about the physical complaints. Twelve respondents with previous musculoskeletal complaints (40.0 %) and 24 respondents with current musculoskeletal complaints (42.9 %) took additional measures to reduce their complaints. Twenty surgeons implemented measures to improve their ergonomics in the operating room. Three surgeons made orthopaedic adjustments and/or used foot pedals less frequently in order to enhance their posture while operating. Seven surgeons accomplished this by strengthening their core muscles by exercising (more frequently) or yoga. Two surgeons are wearing a (night) brace to relieve the tension on the elbow/wrist, and one uses an exoskeleton while operating. Seven paid additional attention to the ergonomics during surgery, such as optimizing the table height, placement of the monitors, and the ability to sit while operating. Other measures included: preventive therapies such as medication or therapy (*n* = 15), temporary decrease in operating hours (*n* = 4), temporarily performing other types of operations (*n* = 5), or even a complete switch to robotic surgery (*n* = 1). The latter respondent suffered from neck and back complaints and has not experienced any physical complaints after this switch.

Out of the 86 surgeons who reported musculoskeletal complaints, 15 (8 with past and 7 with current complaints) needed to completely deposit all their surgical activities for at least one period. Leading to a temporary sick leave rate of 12.7 %, the highest incidence of sick leave was among respondents with neck and/or lower back (43.9 %). The sick leave period had a median of eight (1–30) weeks. These surgeons had a median VAS of 4 (1–10). Eight surgeons (53.3 %) used analgesics, of which four respondents used two or more different analgesics. Four surgeons combined their analgesics with therapy. Three surgeons only used therapy to reduce their complaints.

### Minimal invasive versus open surgery

There were 74 respondents (62.7 %) who performed more endoscopic surgeries than open surgeries, 33 respondents (28.0 %) who performed more open surgeries than endoscopic surgeries, 9 respondents (7.6 %) performed an equal number of endoscopic and open surgeries, and 2 respondents (1.7 %) performed most surgeries with the robot (Table 4). There was no significant difference in characteristics between the endoscopic and open surgery group, except for gender (*p* = 0.047). The median operating hours were 16.0 hours per week (3.0–45.0) for the more endoscopic surgery group and 15.0 hours per week (8.0–30.0) for the more open surgery group (*p* = 0.437). There were no significant difference between the number of previous or current complaints between both groups (*p* = 0.554). The most frequently reported localization of musculoskeletal complaints in the more endoscopic surgery group was the neck (46.3 %), followed by the erector spinae (27.8 %), the right deltoid muscle (16.7 %), and the right latissimus dorsi (13.0 %). The most frequent reported localization of complaints for the more open group was slightly different: the erector spinae (40.9 %), followed by the right latissimus dorsi (31.8 %), the neck (27.3 %), and the right deltoid muscle (18.2 %). In order to reduce their complaints, 27.8 % of the more endoscopic surgery group used analgesics versus 22.7 % of the more open surgery group (*p* = 0.778).

**Table 4:** Respondent characteristics between respondents who perform more endoscopic surgeries versus respondents who perform more open surgeries

Variable	More endoscopic (*n* = 74)	More open (*n* = 33)	*p* value
Sex (male)	46 (62.2 %)	27 (81.8 %)	0.047
Age (years)	47.0 (35.0–62.0)	50.0 (37.0–63.0)	0.056
Height (cm)	180.0 (156.0–204.0)	182.0 (165.0–198.0)	0.130
Weight (kg)	79.0 (52.0–118.0)	81.0 (53.0–113.0)	0.299
Specialism			0.803
General surgeon	58 (78.4 %)	23 (69.7 %)	
Urologist	5 (6.8 %)	3 (9.1 %)	
Gynaecologist	9 (12.2 %)	6 (18.2 %)	
Paediatric surgeon	2 (2.7 %)	1 (3.0 %)	
Hospital (academic/district)	26/48 (35.1 %/64.9 %)	11/22 (33.3 %/66.6 %)	1.000
Dominant hand			0.737
Right	63 (85.1 %)	29 (87.9 %)	
Left	5 (6.8 %)	1 (3.0 %)	
Ambidextrous	6 (8.1 %)	3 (9.1 %)	
Glove size	7.5 (6.0–9.0)	7.5 (6.5–8.5)	0.424
Years of practice	13.0 (0.0–33.0)	16.0 (2.0–30.0)	0.635
Medical history			0.657
None	47 (63.5 %)	25 (75.8 %)	
Operating hours	16.0 (3.0–45.0)	15.0 (8.0–30.0)	0.437
>3 hours	2.0 (0.0–4.0)	2.0 (0.0–6.0)	0.258
Complaints^a^	54 (73.0 %)	22 (66.7 %)	0.500
Localizations^a^			
Neck	25 (46.3 %)	6 (27.3 %)	0.198
Erector spinae	15 (27.8 %)	9 (40.9 %)	0.278
Right deltoid muscle	9 (16.7 %)	4 (18.2 %)	1.000
Right latissimus dorsi	7 (13.0 %)	7 (31.8 %)	0.099
VAS	4.0 (1.0–10.0)	4.0 (2.0–8.0)	0.721
Treatment^b^	13 (24.1 %)	8 (36.4 %)	0.789
Sick leave	9 (16.7 %)	2 (9.1 %)	0.494

Continuous variables are depicted as median (range). Categorical variables are depicted as absolute values (%)

^a^Reported complaints, current and in the past

^b^Therapy: e.g. massage therapy or physical therapy

## Discussion

This cross-sectional national study gains insight into the current state of musculoskeletal injuries and subsequent frequency and duration of sick leave among Dutch surgeons, gynaecologists and urologists. A total of 72.9 % of the respondents is currently suffering or has previously suffered from musculoskeletal complaints. The latter group does not have complaints anymore. Respondents’ most frequently reported complaints were pain in the neck, the (lower) back, and shoulders. The complaints occurred most frequently during surgery. Respondents with a positive medical history for musculoskeletal complaints were more likely to report current or previous complaints. Of the respondents with previous complaints, 40 % had used analgesics or therapies to reduce their complaints and 26.7 % needed to take a sick leave. Currently, 37.5 % of the respondents use analgesics or therapies, and 12.5 % is on sick leave.

### Current state versus previous state

Previous studies addressed the problem considering ergonomics among surgeons in the Netherlands. In order to assess the ergonomics during surgery, different methods were used, such as analysing postures, determining the muscular strain by using electromyography, reporting about the VAS score associated with certain positions, or measuring the angles of certain body parts [10, 20, 21, 23]. Two other Dutch studies, by Wauben et al. [32] and Sari et al. [33], did report on physical complaints and also demonstrated that most physical complaints concerned the neck, shoulders, and back. Unfortunately, only limited information on demographic features was available in these studies, and nothing was said about the need of analgesics and/or therapy, sick leave, or coherence of localizations of musculoskeletal complaints. In addition, the respondents were deemed full laparoscopic surgeons; however, they also performed open surgical procedures [33]. Therefore, the conclusions do not reflect full laparoscopic surgeons. However, the prevalence of physical complaints among these surgeons shown in Sari et al. [33] was comparable to ours. Our more elaborated questionnaire regarding this issue has provided more insight, and the next step will be to tackle the reported ergonomic problems during surgery.

### Minimal invasive versus open surgery

Previous studies have suggested that surgeons experience more musculoskeletal discomfort during minimally invasive surgery [2, 5, 13, 40]. However, we did not observe a higher number of musculoskeletal complaints among surgeons who perform more endoscopic surgeries compared with surgeons who perform more open surgeries. Respondents in both groups seemed to suffer of complaints (now or in the past). This discrepancy could, for example, be due to the fact that other studies categorize surgeons as solely laparoscopic surgeons when they are regularly involved in laparoscopic surgeries [2] or because of the variance in prior surgical experience [40]. This could lead to a higher number of surgeons categorized as ‘laparoscopic’, with musculoskeletal complaints. In our study, surgeons were divided into categories of most frequently used operating technique. According to our definition, surgeons in the more open surgery group could also be regularly involved in laparoscopic surgeries.

### Other studies

Respondents’ most frequently reported symptoms were: discomfort in neck, shoulders, and (lower) back, which was in line with previous studies reporting on intraoperative adjustments and/or sick leave due to musculoskeletal complaints [7, 26, 29, 33, 41]. These localizations are in agreement with the posture during minimally invasive surgery. Surgeons’ static pose during minimally invasive operations resulted in increased muscle fatigue, especially in the neck and back [11, 13]. Additionally, insufficient monitor position causes cervical torsion and results in complaints considering the cervical spine and the upper extremities, including the shoulders [19, 20, 23]. Studies have shown that laparoscopic instruments, which are longer than instruments used in open surgery, cause abduction of the arms during manipulation, also resulting in complaints of the neck and shoulders [42, 43]. Furthermore, the higher incidence of musculoskeletal complaints recorded in other studies could be explained by the fact that these studies asked for incidental injury/illness symptom after a laparoscopic procedure, while we asked for complaints that persisted (now or in the past) [2, 27].

Other studies noted that individuals with a small surgical glove size seem to experience more difficulty in the use of laparoscopic surgical instruments [2, 44], since laparoscopic instruments have no consideration for the varying sizes of hands who use them [45]. They found that women were more likely to report a smaller glove size in comparison with men and were therefore more prone to report discomfort due to their surgical practice [2, 44]. Furthermore, Berguer and Hreljac [44] mentioned that there is also a possibility that large-handed surgeons experience difficulties with the mismatch between instrument size and hand size. As opposed to these studies, we did not find any significant differences in gender, regarding glove size or height, nor did we find significant differences considering their complaints.

Workers in general with sleep problems have been linked to increased risk for occupational injury [46]. The American Academy of Sleep Medicine (AASM) and Sleep Research Society (SRS) recommended that adults should obtain seven or more hours of sleep per night to avoid the health risks of chronic inadequate sleep [47]. According to this standard, only 50 % of all our responding surgeons obtain adequate number of sleeping hours. Our results are consistent with previous studies showing that large numbers of surgeons do not obtain the recommended number of sleeping hours [17]. However, it could not be identified as a risk factor for occupational injury among our respondents, since all surgeons obtain inadequate number of sleeping hours. As opposed to this previous study, number of exercising hours was not identified as a risk factor for occupational injury [17].

As far as we know, this is the first study that reports on medical history of respondents. There was a significant association between respondents with a positive medical history for musculoskeletal complaints and the occurrence of previous or current complaints. Despite the relatively small number of respondents, a positive medical history for musculoskeletal complaints was more frequent reported by surgeons with musculoskeletal complaints in certain localizations. Unfortunately, details of the duration, occurrence, and follow-up of these complaints reported in the medical history are unknown. That is why nothing can be said about whether being a surgeon may have been of influence on any of these complaints.

### Strength and limitations

This study has several strengths and limitations worth mentioning. Firstly, the strength of this study is that this is a more extensive survey with the possibility of defining pain locations more accurately, with the ability of reporting more than one musculoskeletal complaint, and with additional questions on medical history, measures to reduce complaints, and sick leave. However, a survey is a subjective tool in order to assess self-reported musculoskeletal complaints and respondents may not have recalled all complaints in detail. Another limitation might be that surgeons, gynaecologists, and urologists who did not experience any complaints have not filled out the survey, leading to a possible overestimation of the number of musculoskeletal complaints. Furthermore, our sample size is relatively small to make firm statements regarding the risk for musculoskeletal complaints. Lastly, this study does not address whether ergonomic interventions have limited any of the reported complaints.

### Ergonomic interventions to be considered

Previous studies have shown that the most effective way to avoid ergonomic problems is a neutral body posture [6, 23]. Several adaptations have contributed to this, such as endoscopic instruments that have been redesigned [18], the adjustability of the table height [21, 22], and the optimal placement of the monitor [19, 20]. Combining these features will avoid a non-neutral posture in surgeons. In addition, when operating with two surgeons, small adjustments can be made for the assisting surgeon to obtain a neutral position as well; by placing a second monitor and, if there is a mismatch in height, add a small bench for one of the two surgeons. Furthermore, various forms of exercise in order to strengthen the core muscles can improve the physical condition. In addition, the musculoskeletal system can be relieved by the use of orthopaedic foot gear, a brace, or even an exoskeleton. Depending on a surgeon’s individual complaint(s), aforementioned adaptations can be considered to achieve an ergonomic body posture.

In the past, various studies have been conducted to evaluate musculoskeletal complaints among surgeons. Several recommendations have been made to reduce complaints. Nevertheless, occupational musculoskeletal complaints among surgeons still exist. Surgeons with a positive medical history seem to report a higher occurrence of previous or current musculoskeletal complaints. Respondents’ most frequently reported complaints were pain in the neck, the (lower) back, and shoulders. Most of the musculoskeletal complaints were only present while performing surgical procedures. No significant difference was found when comparing respondents who performed more endoscopic and more open surgery. Surgeons used analgesics and/or therapy to reduce their complaints, and even sick leave was necessary to fully recover. About half of the respondents with complaints made ergonomic adjustments in the operating room to prevent future complaints. The latter would be interesting for future research to develop a programme that could reduce musculoskeletal complaints and associated sick leave during a surgeon’s career.
